# Liver Stiffness Measurement-Based Scoring System for Significant Inflammation Related to Chronic Hepatitis B

**DOI:** 10.1371/journal.pone.0111641

**Published:** 2014-10-31

**Authors:** Mei-Zhu Hong, Ru-Mian Zhang, Guo-Liang Chen, Wen-Qi Huang, Feng Min, Tian Chen, Jin-Chao Xu, Jin-Shui Pan

**Affiliations:** 1 Department of Infectious Diseases, Chenggong Hospital affiliated to Xiamen University (the 174th Hospital of PLA), Xiamen, Fujian, China; 2 Hepatology Unit and Department of Infectious Diseases, Xiamen Hospital of Traditional Chinese Medicine, Xiamen, Fujian, China; 3 Department of Gastroenterology, Zhongshan Hospital affiliated to Xiamen University, Xiamen, Fujian, China; University of Pisa, Italy

## Abstract

**Objectives:**

Liver biopsy is indispensable because liver stiffness measurement alone cannot provide information on intrahepatic inflammation. However, the presence of fibrosis highly correlates with inflammation. We constructed a noninvasive model to determine significant inflammation in chronic hepatitis B patients by using liver stiffness measurement and serum markers.

**Methods:**

The training set included chronic hepatitis B patients (n = 327), and the validation set included 106 patients; liver biopsies were performed, liver histology was scored, and serum markers were investigated. All patients underwent liver stiffness measurement.

**Results:**

An inflammation activity scoring system for significant inflammation was constructed. In the training set, the area under the curve, sensitivity, and specificity of the fibrosis-based activity score were 0.964, 91.9%, and 90.8% in the HBeAg(+) patients and 0.978, 85.0%, and 94.0% in the HBeAg(−) patients, respectively. In the validation set, the area under the curve, sensitivity, and specificity of the fibrosis-based activity score were 0.971, 90.5%, and 92.5% in the HBeAg(+) patients and 0.977, 95.2%, and 95.8% in the HBeAg(−) patients. The liver stiffness measurement-based activity score was comparable to that of the fibrosis-based activity score in both HBeAg(+) and HBeAg(−) patients for recognizing significant inflammation (G ≥3).

**Conclusions:**

Significant inflammation can be accurately predicted by this novel method. The liver stiffness measurement-based scoring system can be used without the aid of computers and provides a noninvasive alternative for the prediction of chronic hepatitis B-related significant inflammation.

## Introduction

Histological information provided by liver biopsy is critical for prognosis evaluation and decision making on antiviral treatment for patients with chronic hepatitis B (CHB). Key histological information includes the stage of fibrosis, the grade of inflammation, and presence of accompanying diseases. Therefore, histological examination of the liver biopsy is regarded by several guidelines as an integral part of the pretreatment evaluation of CHB patients [1]–[3]. However, due to the invasiveness, not all patients accept to undergo liver biopsy. Thus, the noninvasive marker elevated serum alanine aminotransferase (ALT) level is conventionally considered the most important factor when choosing antiviral treatment in CHB, even if the ALT level is not always consistent with intrahepatic inflammation. On the other hand, patients with persistently normal serum ALT levels may not accept to undergo liver biopsy even though such patients are at risk of progressing to advanced fibrosis or cirrhosis [4]. Sampling error and interobserver variability also hinder liver biopsy from being applied more widely [5].

In the last decade, transient elastography has emerged as an accurate and noninvasive tool to detect diverse stages of liver fibrosis or liver cirrhosis [6]–[9]. Transient elastography (FibroScan) has been proposed for the assessment of liver fibrosis by using liver stiffness measurement (LSM). According to several studies, the area under the curve (AUC) of LSM for differentiating moderate fibrosis (S ≥2) and cirrhosis (S = 4) is 0.80–0.88 and 0.94–0.99, respectively, suggesting that LSM has an excellent performance in the diagnosis of fibrosis (Table S1) [6]–[10]. Moreover, the result of LSM is reproducible [11]. Despite the mentioned advantages such as accuracy, noninvasiveness, and reproducibility, LSM cannot replace liver biopsy currently, mainly because it does not provide information on intrahepatic inflammation. However, information on inflammatory activity is important when choosing antiviral therapy and in predicting the response of antiviral therapy, especially after interferon-based treatment. Patients with high activity tend to have high rates of anti-HBe seroconversion [3].

Several models have been generated for the prediction of inflammatory activity in CHB [4], [12]–[14] and chronic hepatitis C [15], [16]. According to our previous research, the stage of fibrosis is highly correlated with the grade of inflammation [17]. Multivariate analysis in a previous study has also revealed that the stage of fibrosis is highly predictive of significant inflammation [14]. Moreover, several fibrosis-related serum markers such as hyaluronate and procollagen III N-terminal peptide (PIIINP) are also useful for predicting inflammation [12]–[14]. It seems reasonable to deduct that the correlation between fibrosis and inflammation acts as a “bridge” connecting fibrosis-related serum markers to inflammation. Elasticity is physical measure resulting by the combination of 3 vectors or determinant factors for the reduced elasticity of the liver: congestion, inflammation and fibrosis. Research by Coco *et al*. firstly demonstrate the impact of necro-inflammation on the elasticity score [18]. Therefore, FibroScan may be valuable for predicting intrahepatic inflammation. The present study aimed to evaluate the diagnostic accuracy of FibroScan combined with serum markers to predict inflammatory activity in a noninvasive way and to make a comparison with other researches.

## Methods

### Patients

Treatment-naïve CHB patients referred to the 174^th^ Hospital of the PLA in Fujian, China, between 2010 and 2013, were enrolled in the training set. Patients referred to the Zhongshan Hospital Affiliated to Xiamen University and Xiamen Hospital of Traditional Chinese Medicine were enrolled in the validation set. Patients in the training set were investigated retrospectively, whereas the constructed algorithm was tested in a prospective manner for the validation set. Patients in the validation set also underwent LSM. The present study was approved by the ethics committees of Zhongshan Hospital affiliated to Xiamen University, Xiamen Hospital of Traditional Chinese Medicine, Chenggong Hospital affiliated to Xiamen University. All patients provided written consent prior to liver biopsy and study entry. Liver biopsies were performed on the same day that serum samples were collected or less than 2 days thereafter. Significant inflammation was defined as having Grade 3–4 inflammation according to the liver biopsy results [19]. This study was conducted according to the principles of the Declaration of Helsinki.

Participants were recruited according to the status of serum HBV markers. Inclusion criteria for patients were: HBsAg-positive with scheduled liver biopsy, regardless of HBeAg status. Patients were excluded if they had the following: Hepatitis C virus, hepatitis D virus, or human immunodeficiency virus co-infection; malnutrition; significant steatosis; alcoholic fatty liver; and decompensated cirrhosis. The study population was a consecutive series of participants defined by the selection criteria. The participants in training set and validation set were enrolled at a ratio of 3∶1. A total of 327 treatment-naïve CHB patients were included in the training set and 106 patients were enrolled to the validation set.

### Diagnostic tests

Hepatitis B virus (HBV) DNA levels were determined by quantitative fluorescence polymerase chain reaction on an ABI 7000 (Applied Biosystems, Carlsbad, USA), with a lower limit of detection of 500 IU/mL. HBV DNA was expressed as log IU/mL. Serum ALT and aspartate aminotransferase (AST) levels were expressed as IU/L. Albumin, γ-glutamyl transpeptidase (GGT), cholinesterase, globulin, urea nitrogen, creatinine, and pre-albumin were tested by chemistry analyzer TBA-120FR (Toshiba, Tochigi, Japan). Liver histology was assessed by using the Scheuer scoring system [19]. Liver histology was evaluated by two independent pathologists who were blind to the study design. If the two pathologists could not agree on the pathological diagnosis, histological scores were calculated and confirmed by a panel of pathologists. Patients were separated into two groups by Scheuer scale G = 0, 1, 2 or G = 3, 4. LSM was performed by using FibroScan according to the training provided by the manufacturer, and was assayed by two well-trained physicians. Liver stiffness was expressed in kPa. Ten successful acquisitions were obtained and the ratio of interquartile range over LSM was lower than 0.3.

### Data analysis

Statistical analyses were performed using IBM SPSS 21 (SPSS Inc., Chicago, IL, USA) or GraphPad Prism 6.01 (GraphPad Software Inc., La Jolla, CA, USA). Quantitative data conformed to Gaussian distribution were tested using analysis of variance. Qualitative data or quantitative data that did not pass a Gaussian distribution test were analyzed using a nonparametric test. All tests were two-sided, and *p*<0.05 was regarded as statistically significant. HBV DNA was logarithmically transformed. The Gini index based on random forest was used to determine whether the identified variables were associated with the grade of intrahepatic inflammation. Gini index based on random forest is widely used in bioinformatics and can be employed in the screening of potential variables that have significant contribution to the independent variable [17], [20], [21].

## Results

### Patient characteristics

Among the 327 patients enrolled in the training set, 190 (58.1%) were HBeAg(+) and 137 (41.9%) were HBeAg(−) (Table S2). In the training set, the grade of inflammation activity observed in HBeAg(+) patients was significantly higher than that in HBeAg(−) patients (median: 2 vs. 1, *p* = 0.0139). Both the training and validation sets had more men than women. In both sets, the mean ALT and AST levels were significantly higher in HBeAg(+) patients than in HBeAg(−) patients (*p*<0.0001). The serum levels of HBV DNA were significantly higher in HBeAg(+) patients than in HBeAg(−) patients, in both sets (*p*<0.0001). Patients in the two sets had similar baseline characteristics. Fifty-eight (17.7%) and 43 patients (40.2%) in the training and validation cohort, respectively, had significant inflammation (G ≥3). No severe complication occurred during or after liver biopsy.

### Factors associated with significant inflammation in the training set

As shown in Table 1, patients with significant inflammation tended to have a higher stage of fibrosis and higher ALT, AST, and GGT levels, whereas they had lower cholinesterase, albumin, and pre-albumin levels. In the HBeAg(+) patients, the Gini index screened out by random forest showed that the factors associated with significant inflammation were stage of fibrosis, GGT, pre-albumin, AST, albumin, cholinesterase, ALT, globulin, and HBV DNA (log_10_ IU/mL), listed according to the contribution, from highest to lowest, to the prediction of significant inflammation (Table S3). In the HBeAg(−) patients, the ranking of associated factors were stage of fibrosis, cholinesterase, pre-albumin, GGT, albumin, AST, ALT, globulin, and HBV DNA (log_10_ IU/mL) (Table S3).

**Table 1:** Characteristics of the patients with significant inflammation in the training set.

	HBeAg(+) (n = 190)		HBeAg(−) (n = 137)	
	G = 0, 1, 2	G = 3, 4	G = 0, 1, 2	G = 3, 4
	(n = 153)	(n = 37)	(n = 117)	(n = 20)
Age, y	32.0±8.2	34.5±10.0	40.6±9.5	38.7±10.8
Men, n (%)	119 (77.8)	26 (70.3)	94 (80.3)	17 (85.0)
ALT, IU/L	128.5±129.0	371.1±430.1	73.7±69.2	267.2±310.5
Normal ALT, n (%)	19 (12.4)	0 (0.0)	39 (33.3)	2 (10.0)
AST, IU/L	66.4±62.7	206.2±260.6	43.4±44.1	130.0±124.7
Normal AST, n (%)	65 (42.5)	4 (10.8)	81 (69.2)	4 (20.0)
CHE, IU/L	8500±1600	6900±1400	8900±1700	6500±1300
GGT, IU/L	42.3±38.9	95.9±62.4	35.9±25.2	122.4±100.9
Globulin, g/L	27.9±3.7	29.9±4.5	27.3±4.2	29.0±4.2
Albumin, g/L	45.1±3.1	42.7±2.7	45.0±2.6	40.9±3.5
Pre-albumin, mg/L	236.7±63.8	158.0±45.4	253.1±60.9	167.2±52.4
Fibrosis*, n (%)				
S0	19 (12.4)	0 (0.0)	31 (26.5)	0 (0.0)
S1	86 (56.2)	0 (0.0)	47 (40.2)	0 (0.0)
S2	37 (24.2)	13 (35.1)	27 (23.1)	4 (20.0)
S3	10 (6.5)	21 (56.8)	8 (6.8)	10 (50.0)
S4	1 (0.7)	3 (8.1)	4 (3.4)	6 (30.0)
HBV DNA, log_10_ IU/mL	7.10±1.55	6.81±1.36	4.55±1.54	4.93±1.83

*According to the Scheuer scoring system.

Abbreviations: ALT, alanine aminotransferase; AST, aspartate aminotransferase; GGT, γ-glutamyl transpeptidase; CHE, cholinesterase; HBV, hepatitis B virus.

### Contributions of the associated factors to significant inflammation in the training set

**Figure 1:**
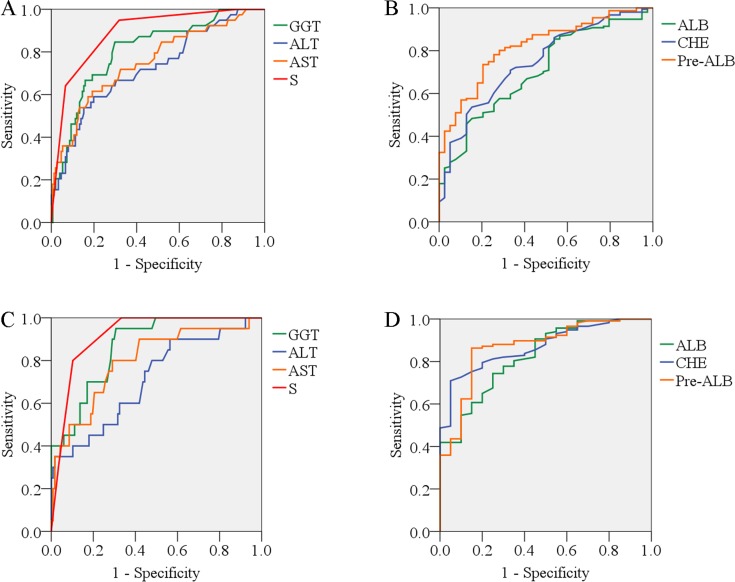
Area under the curve (AUC) of the enrolled variables in differentiating significant inflammation in the training set. (A) AUC of γ-glutamyl transpeptidase (GGT), alanine aminotransferase (ALT), aspartate aminotransferase (AST), and fibrosis (S) in the HBeAg(+) patients; (B) AUC of albumin (ALB), cholinesterase (CHE), and pre-albumin (Pre-ALB) in the HBeAg(+) patients; (C) AUC of GGT, ALT, AST, and fibrosis (S) in the HBeAg(−) patients; (D) AUC of ALB, CHE, and Pre-ALB in the HBeAg(−) patients.

We performed AUC analysis to indicate the efficacy of the associated factors on the prediction of significant inflammation. As shown in Table 2, both in the HBeAg(+) and HBeAg(−) patients, the stage of fibrosis had the greatest AUC. Contributions of other associated factors were shown in Table 2 and Fig. 1A∼1D. The ranking of associated factors listed by AUC from highest to lowest was in accordance to the sequence of associated factors listed by Gini index (Table S3). By performing AUC analysis, we also confirmed the optimal cut-off value of each associated factor (Table 2). The prediction performance of each cut-off value was also calculated (Table 2).

**Table 2:** Diagnostic performance of the enrolled variables in differentiating significant inflammation in the training set.

Variable	AUC	95% CI	Cut-off	Sensitivity (%)	Specificity (%)
HBeAg (+)					
Fibrosis*	0.890	0.835–0.944	S ≥2	95.1	68.2
Pre-albumin	0.819	0.752–0.887	180 mg/L	78.4	81.0
GGT	0.806	0.729–0.882	56 IU/L	73.0	81.0
AST	0.752	0.661–0.843	75 IU/L	65.9	75.2
CHE	0.747	0.665–0.829	7600 IU/L	67.6	72.5
ALT	0.723	0.630–0.816	125 IU/L	65.9	68.6
Albumin	0.706	0.620–0.792	43 g/L	64.9	79.1
HBeAg (−)					
Fibrosis*	0.917	0.868–0.966	S ≥2	100.0	66.7
CHE	0.870	0.802–0.938	7500 IU/L	75.0	81.2
Pre-albumin	0.869	0.783–0.954	195 mg/L	80.0	86.3
GGT	0.865	0.793–0.937	52 IU/L	70.0	82.1
Albumin	0.826	0.734–0.919	43 g/L	70.0	77.8
AST	0.797	0.687–0.908	46 IU/L	65.0	77.8
ALT	0.710	0.582–0.839	72 IU/L	60.0	67.5

*According to the Scheuer scoring system.

Abbreviations: Abbreviations: AUC, area under the curve; CI, confidence interval; GGT, γ-glutamyl transpeptidase; AST, aspartate aminotransferase; CHE, cholinesterase; ALT, alanine aminotransferase.

### Construction of the prediction score

**Figure 2:**
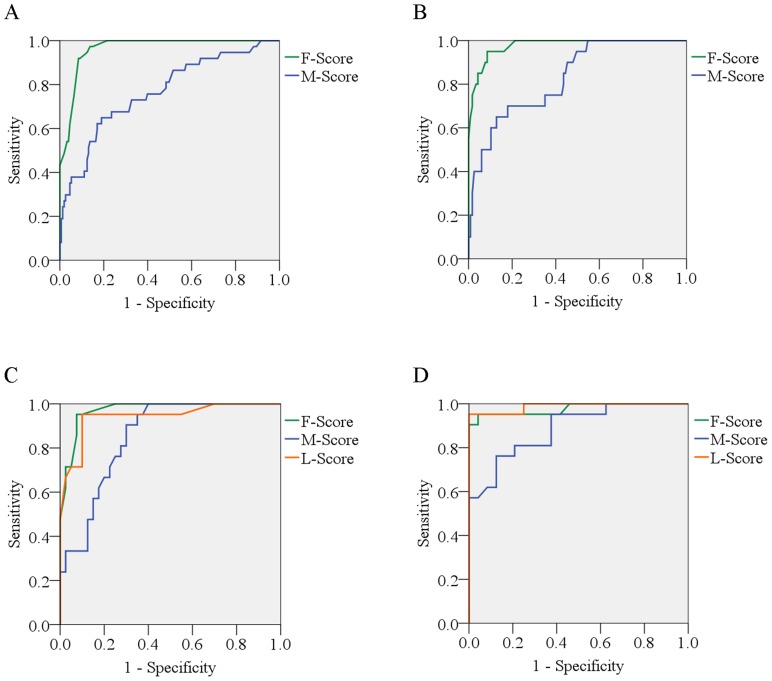
Area under the curve (AUC) of the fibrosis-based activity score (F-score), Mohamadnejad *et al.* score (M-score), and LSM-based activity score (L-score) in differentiating significant inflammation. (A) AUC of the F-Score and M-Score for the HBeAg(+) patients in the training set; (B) AUC of the F-Score and M-Score in the HBeAg(−) patients in the training set; (C) AUC of the F-Score, M-Score, and L-Score in the HBeAg(+) patients in the validation set; (D) AUC of the F-Score, M-Score, and L-Score in the HBeAg(−) patients in the validation set.

A fibrosis-based prediction score was constructed according to its relative contribution, as determined by: 1/(1-AUC) (Table 3). For convenience, the factor with the lowest score was set as 10 whereas other factors were rounded to the nearest integers by calibrating their 1/(1-AUC) with the variable with the lowest 1/(1-AUC), respectively. The calculation of fibrosis-based score was shown in Table S4. Final score of each patient was the cumulative score of the respective score of enrolled independent variable in this patient. This new fibrosis-based activity score ranged from −70 to 70 in the HBeAg(+) patients, and from −93 to 93 in the HBeAg(−) patients. The AUC was 0.964 (95% confidence interval (CI), 0.940–0.987; *p*<0.0001; Fig. 2A) in HBeAg(+) patients and 0.971 (95% CI, 0.937–1.000; *p*<0.0001; Fig. 2B) in HBeAg(−) patients.

**Table 3:** Prediction model of significant inflammation.

	HBeAg(+)		HBeAg(−)	
Factors	Cut-off	Score	Cut-off	Score
Fibrosis*	S ≥2	+23	S ≥2	+24
	S <2	−23	S <2	−24
Pre-albumin	≥180 mg/L	−14	≥195 mg/L	−16
	<180 mg/L	+14	<195 mg/L	+16
GGT	≥56 IU/L	+13	≥52 IU/L	+15
	<56 IU/L	−13	<52 IU/L	−15
AST	≥75 IU/L	+10	≥46 IU/L	+10
	<75 IU/L	−10	<46 IU/L	−10
CHE	≥7600 IU/L	−10	≥7500 IU/L	−16
	<7600 IU/L	+10	<7500 IU/L	+16
Albumin			≥43 g/L	−12
			<43 g/L	+12

*According to the Scheuer scoring system.

Abbreviations: GGT, γ-glutamyl transpeptidase; AST, aspartate aminotransferase; CHE, cholinesterase.

Using 10 as the cut-off value, the sensitivity and specificity were 91.9% (34/37) and 90.8% (139/153), respectively, in HBeAg(+) patients (Table 4). In HBeAg(−) patients, the sensitivity and specificity were 85.0% (17/20) and 94.0% (110/117), respectively, when a cutoff value of 12 was applied (Table 4). In HBeAg(+) patients, activity scores of the patients with significant inflammation (G 3, 4) were markedly higher than that of the patients without inflammation (G 0) or with mild (G 1) or moderate inflammation (G 2) (Kruskal-Wallis test, *p*<0.0001; Fig. S1A). Similar phenomena was also observed in HBeAg(−) patients (Kruskal-Wallis test, *p*<0.0001; Fig. S1B).

**Table 4:** Efficacy of the prediction model on significant inflammation.

		HBeAg(+)		HBeAg(−)	
		Training set	Validation set	Training set	Validation set
Fibrosis-Activity Score	AUC	0.964	0.971	0.978	0.977
	95% CI	0.940–0.987	0.937–1.000	0.954–1.000	0.935–1.000
	Cut-off	10	10	12	12
	Sensitivity (%)	91.9	90.5	85.0	95.2
	Specificity (%)	90.8	92.5	94.0	95.8
	PPV (%)	70.8	86.4	70.8	95.2
	NPV (%)	97.9	94.9	97.3	95.8
LSM-Activity Score	AUC		0.942		0.988
	95% CI		0.877–1.000		0.963–1.000
	Cut-off		10		12
	Sensitivity (%)		90.5		95.2
	Specificity (%)		90.0		95.8
	PPV (%)		82.6		95.2
	NPV (%)		94.7		95.8
Mohamadnejad’s. Score	AUC	0.767	0.849	0.829	0.886
	95% CI	0.677–0.856	0.757–0.942	0.737–0.921	0.791–0.980
	Cut-off	6.36	6.36	7.54	7.54
	Sensitivity (%)	70.3	94.5	70.0	81.0
	Specificity (%)	68.0	70.0	81.2	70.8
	PPV (%)	34.7	61.3	38.9	70.8
	NPV (%)	90.4	93.3	94.1	81.0

Abbreviations: AUC, area under the curve; CI, confidence interval; PPV, positive predictive value; NPV, negative predictive value; LSM, liver stiffness measurement.

### Validation of activity score

In the validation set, the AUC, sensitivity, and specificity were 0.971 (95% CI, 0.937–1.000), 90.5% (19/21), and 92.5% (37/40) for HBeAg(+) patients and 0.977 (95% CI, 0.935–1.000), 95.2% (20/21), and 95.8% (23/24) for HBeAg(−) patients, respectively (Table 4). Since LSM is an accurate index for the diagnosis of moderate to severe fibrosis, it seems reasonable to use noninvasive LSM as an alternative to invasive liver sampling. A cut-off value of 7.2 kPa was used for the diagnosis of moderate to severe fibrosis (S≥2) [22]. In the HBeAg(+) patients in the validation set, the AUC of LSM that differentiated patients with fibrosis (S≥2) was 0.938 (95% CI, 0.882–0.994); the sensitivity and specificity were 87.5% and 79.3%, respectively. In the HBeAg(−) patients in the validation set, the AUC was 0.889 (95% CI, 0.793–0.985); the sensitivity and specificity were 89.7% and 75.0%, respectively.

In the validation set, when the patients with LSM≥7.2 kPa were regarded as having S≥2 fibrosis, LSM-based activity scores were calculated for each patient. Regarding the LSM-based activity score, the AUC, sensitivity, and specificity were 0.942 (95% CI, 0.877–1.000), 90.5% (19/21), and 90.0% (36/40) in the HBeAg(+) patients and 0.988 (95% CI, 0.963–1.000), 95.2% (20/21), and 95.8% (23/24) in the HBeAg(−) patients, respectively (Table 4).

### Diagnostic performance of other models

Several models were constructed for the prediction of significant inflammation. Mohamadnejad *et al*. [4] reported that significant inflammation can be predicted by a model consisting of age, HBV DNA level, AST, and albumin. Using our data, the AUC according to the Mohamadnejad *et al.* model was 0.767 and 0.849 in the HBeAg(+) patients in the training and validation sets, respectively (Table 4). Regarding the HBeAg(−) patients in the training and validation sets, the AUC according to the Mohamadnejad *et al.* model was 0.829 and 0.886, respectively (Table 4). The AUCs according to the Mohamadnejad *et al.* model in each set are also shown in Fig. 2. Maybe due to the ethnic difference, the cutoff values used by Mohamadnejad *et al.* cannot work well in our data. In order to get a balanced sensitivity and specificity, 6.36 and 7.54 were used as cutoff values for HBeAg(+) and HBeAg(−) patients, respectively.

## Discussion

In brief, we aimed to construct a predictive model based on stage of fibrosis and serum markers for significant inflammation related to CHB. First, the most distinguishing feature of our scoring system is that the stage of fibrosis or LSM is enrolled as an independent variable for the prediction of inflammation. Second, the diagnosis performance of our scoring algorithm is excellent for both HBeAg(+) and HBeAg(−) patients. It could be expected that if the method of LSM was implemented and the related serum markers were available, over 90% of liver biopsies could be avoided. Third, the scoring system is feasible and ready to use even without the aid of a computer.

In the present study, the weighted score of each variable was calculated by the following formula: 1/(1-AUC). In the formula, AUC represents the percentile of patients in the training set who can be “rightly diagnosed” by a specific variable, whereas “1” represents the total of patients in the training set. Thus, the formula represents the ratio of “total patients” to “wrongly diagnosed patients”. In other words, the ratio tends to be higher in the variable with higher predictive value. Therefore, in our scoring system, the score of each variable is determined by its diagnostic performance. Although the traditional logistic regression method and several modern data mining methods such as random forest can also be used to process similar data, the user cannot perform complex calculations without the aid of a computer and special analysis software. Furthermore, the diagnostic performance cannot be significantly improved if logistic regression and random forest were used (Table S5).

Note that the cut-off value of each variable has a profound effect on the efficacy of the scoring system. In our scoring system, fibrosis (S ≥2) tends to have high sensitivity, whereas the specificity is relatively low. In order to obtain a balanced performance between sensitivity and specificity, we set cut-off values with relatively high specificity for other variables besides fibrosis. Additionally, the number of enrolled variables also has a significant effect on the diagnostic performance of the scoring system. In HBeAg(+) patients, when fibrosis, pre-albumin, GGT, AST, and cholinesterase (CHE) were enrolled, the AUCs of the training and validation sets reached the peak with balanced sensitivity and specificity (Table S6). However, in HBeAg(−) patients, when albumin was added as an independent variable, the diagnostic performance of the scoring system was further improved (Table S6).

Several guidelines recommend that patients who remain HBeAg(+), with HBV DNA levels >20,000 IU/mL accompanied by a mild elevation of ALT levels, should be considered for liver biopsy and treatment should be considered if the biopsy showed moderate or severe inflammation or significant fibrosis [1], [3]. It seems necessary to divide the intrahepatic inflammation into three groups: no inflammation to mild (G 0–1), moderate (G 2), and severe inflammation (G 3–4). However, in our scoring system, intrahepatic inflammation is divided into no inflammation to moderate inflammation (G 0–2) and severe inflammation (G 3–4); that is, the dependent variable is a dichotomy index. There are several reasons to treat the dependent variable this way. First, more suitable methods such as logistic regression and random forest are used to analyze the data with the dependent variable as a dichotomy index. Second, according to our data, there is no dramatic difference between G0–1inflammation and G2 inflammation. In HBeAg(+) patients with S1 fibrosis, there is no difference in ALT, AST, CHE, and pre-albumin levels, except GGT between G 0–1 and G 2 inflammation (Fig. S2). In HBeAg(−) patients with S1 fibrosis, though there is difference in ALT, AST, and GGT levels between G 0–1 and G 2 inflammation, it is comparable for CHE and pre-albumin (Fig. S3). Therefore, we think it is appropriate to divide the grade of inflammation into no to moderate inflammation (G 0–2) and severe inflammation (G 3–4).

In our study, the AUCs of LSM that differentiated patients with fibrosis (S ≥2) (0.938 and 0.889 for the HBeAg(+) and HBeAg(−) patients, respectively) are higher than those reported by Marcellin *et al.* [22] and lower than those reported by Coco *et al* [23]. Note that in the clinical practice, we have found that patients with high body mass index tend to have a higher LSM. A similar phenomenon has also been observed by Castera *et al.* [24] and Cardoso *et al* [25]. A later-generation FibroScan can provide simultaneously controlled attenuation parameter, an index of steatosis. Patients with significant liver steatosis were excluded from the present research.

Compared with the values calculated using the Mohamadnejad *et al.* model, the sensitivity, specificity, positive predictive value, and negative predictive value were greatly improved by using our model. The introduction of stage of fibrosis as an independent variable may have contributed to this improvement. As our previous research has indicated, there is a significant correlation between the grade of inflammation and the stage of fibrosis [17], which is consistent with a study by Cheong *et al* [14]. Several other studies also indicate the relationship between the grade of inflammation and the stage of fibrosis [12], [13]. In order to predict intrahepatic inflammation in a noninvasive manner, we used LSM instead of stage of fibrosis in our model and the diagnostic performance of the LSM-based activity score was comparable to that of the fibrosis-based activity score. Therefore, we can accurately predict significant inflammation with a noninvasive method.

Immunotolerant patients over 30 years of age and/or with a family history of hepatocellular carcinoma or cirrhosis are recommended to consider liver biopsy [3]. Thus, the prediction of significant necroinflammation in this subgroup of patients is of clinical value. The diagnostic performance of our model is also excellent in patients with normal ALT (Table S7).

In conclusion, significant inflammation can be accurately predicted by this novel method. The LSM-based scoring system can be used without the aid of computers and provides a noninvasive alternative for the prediction of CHB-related significant inflammation. For convenience, we have developed a web-based calculator for our scoring system, which is available at http://www.zhhep.com/inflammation.php. All of what the user should do is just select the appropriate options according to his specific condition, and the prediction result will be got. However, the limitation is that only the patients in the validation set have LSM results.

## Supporting Information

Figure S1 **Fibrosis-based activity scores of the patients with different grades of inflammation in the training set.** (A) In the HBeAg(+) patients, the fibrosis-based activity scores of the patients with significant inflammation (G 3, 4) were markedly higher than that of the patients with without (G 0) or with mild (G 1) or moderate inflammation (G 2) (Kruskal-Wallis test, *p*<0.0001); (B) In the HBeAg(−) patients, the fibrosis-based activity scores of the patients with significant inflammation (G 3, 4) were markedly higher than that of the patients with without (G 0) or with mild (G 1) or moderate inflammation (G 2) (Kruskal-Wallis test, *p*<0.0001).
(TIF)

Figure S2 **Levels of the enrolled variables in the HBeAg(+) patients with S1 fibrosis with no to mild or moderate inflammation in the training set.** (A) ALT (Mann Whitney test, *p* = 0.0508); (B) AST (Mann Whitney test, *p* = 0.1022); (C) CHE (Mann Whitney test, *p* = 0.3295); (D) GGT (Mann Whitney test, *p* = 0.0125); (E) pre-ALB (Mann Whitney test, *p* = 0.1803). Abbreviations: ALT, alanine aminotransferase; AST, aspartate aminotransferase; CHE, cholinesterase; GGT, γ-glutamyl transpeptidase; pre-ALB, pre-albumin.
(TIF)

Figure S3 **Levels of enrolled variables in the HBeAg(−) patients with S1 fibrosis with no to mild or moderate inflammation in the training set.** (A) ALT (Mann Whitney test, *p* = 0.0070); (B) AST (Mann Whitney test, *p* = 0.0066); (C) CHE (Mann Whitney test, *p* = 0.7717); (D) GGT (Mann Whitney test, *p* = 0.0464); (E) pre-ALB (Mann Whitney test, *p* = 0.3470). Abbreviations: ALT, alanine aminotransferase; AST, aspartate aminotransferase; CHE, cholinesterase; GGT, γ-glutamyl transpeptidase; pre-ALB, pre-albumin.
(TIF)

Table S1 **Area under the curve for differentiating specific stage of fibrosis (S).**
(DOCX)

Table S2 **Characteristics of the patients enrolled in the training and validation sets.**
(DOCX)

Table S3 **The Gini index of the factors associated with significant inflammation (G).**
(DOCX)

Table S4 **Calculation of Fibrosis-based activity score.**
(DOCX)

Table S5 **Diagnostic performance of logistic regression and random forest for recognizing significant inflammation.**
(DOCX)

Table S6 **Relationship between number of enrolled variables and diagnostic performance of the prediction score.**
(DOCX)

Table S7 **Efficacy of the fibrosis-based activity score in the patients with normal alanine aminotransferase.**
(DOCX)
