# Supplementary figures and images for: Liver Stiffness Measurement-Based Scoring System for Significant Inflammation Related to Chronic Hepatitis B
Source: PLoS One. 2014 Oct 31;9(10):e111641. doi: 10.1371/journal.pone.0111641 (PMC4216134; doi:10.1371/journal.pone.0111641)

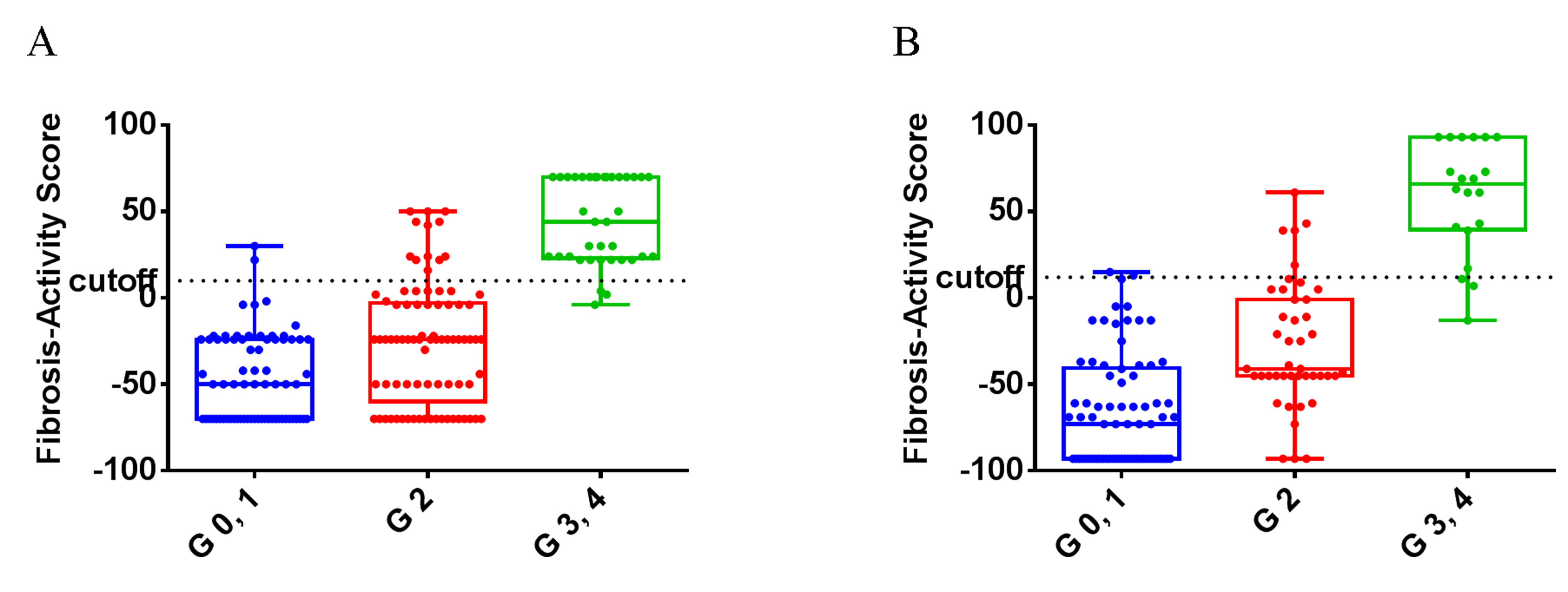

Supplement: Figure S1 — Fibrosis-based activity scores of the patients with different grades of inflammation in the training set. (A) In the HBeAg(+) patients, the fibrosis-based activity scores of the patients with significant inflammation (G 3, 4) were markedly higher than that of the patients with without (G 0) or with mild (G 1) or moderate inflammation (G 2) (Kruskal-Wallis test, p<0.0001); (B) In the HBeAg(−) patients, the fibrosis-based activity scores of the patients with significant inflammation (G 3, 4) were markedly higher than that of the patients with without (G 0) or with mild (G 1) or moderate inflammation (G 2) (Kruskal-Wallis test, p<0.0001). (TIF) [file pone.0111641.s001.tif]

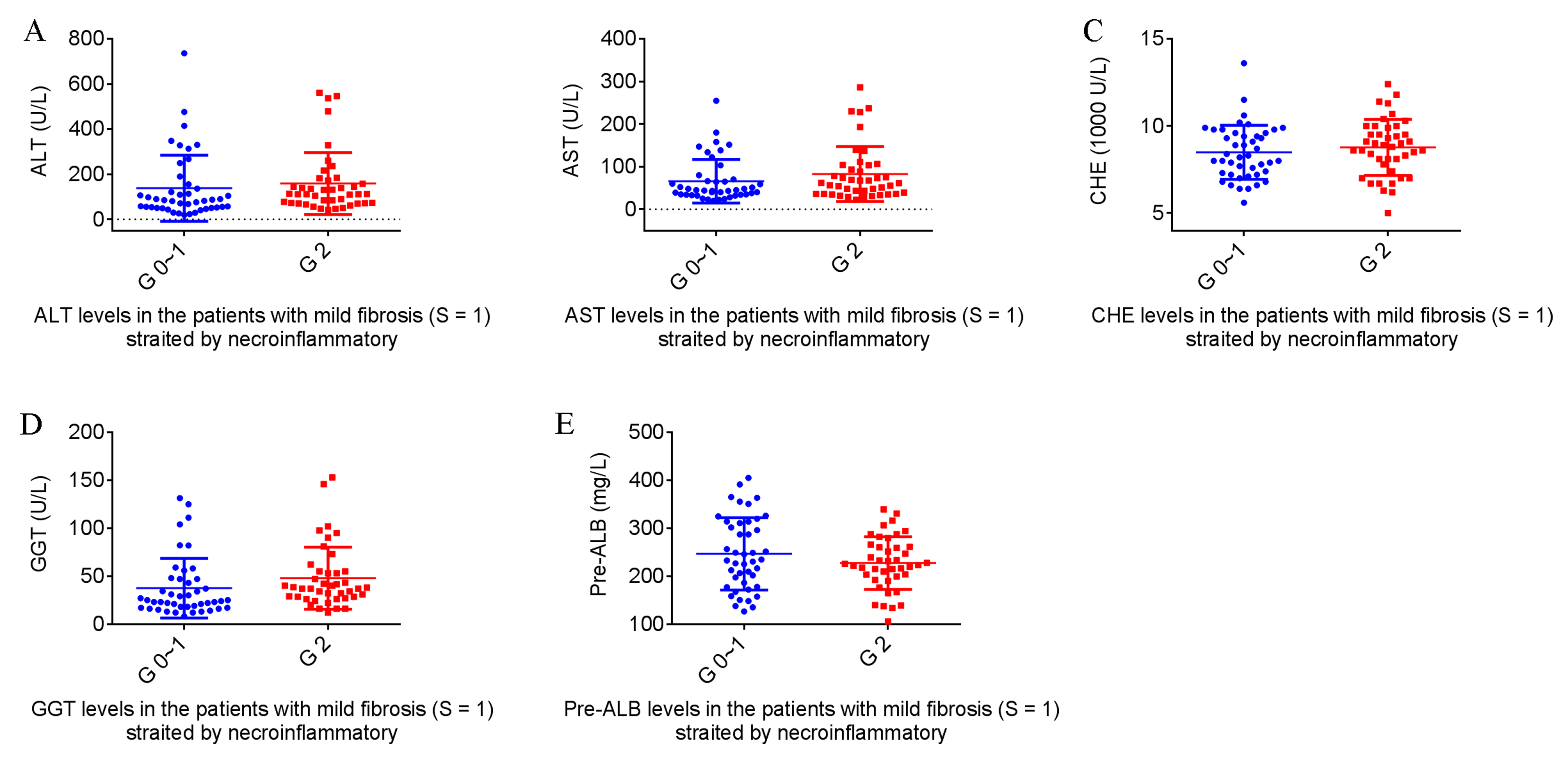

Supplement: Figure S2 — Levels of the enrolled variables in the HBeAg(+) patients with S1 fibrosis with no to mild or moderate inflammation in the training set. (A) ALT (Mann Whitney test, p = 0.0508); (B) AST (Mann Whitney test, p = 0.1022); (C) CHE (Mann Whitney test, p = 0.3295); (D) GGT (Mann Whitney test, p = 0.0125); (E) pre-ALB (Mann Whitney test, p = 0.1803). Abbreviations: ALT, alanine aminotransferase; AST, aspartate aminotransferase; CHE, cholinesterase; GGT, γ-glutamyl transpeptidase; pre-ALB, pre-albumin. (TIF) [file pone.0111641.s002.tif]

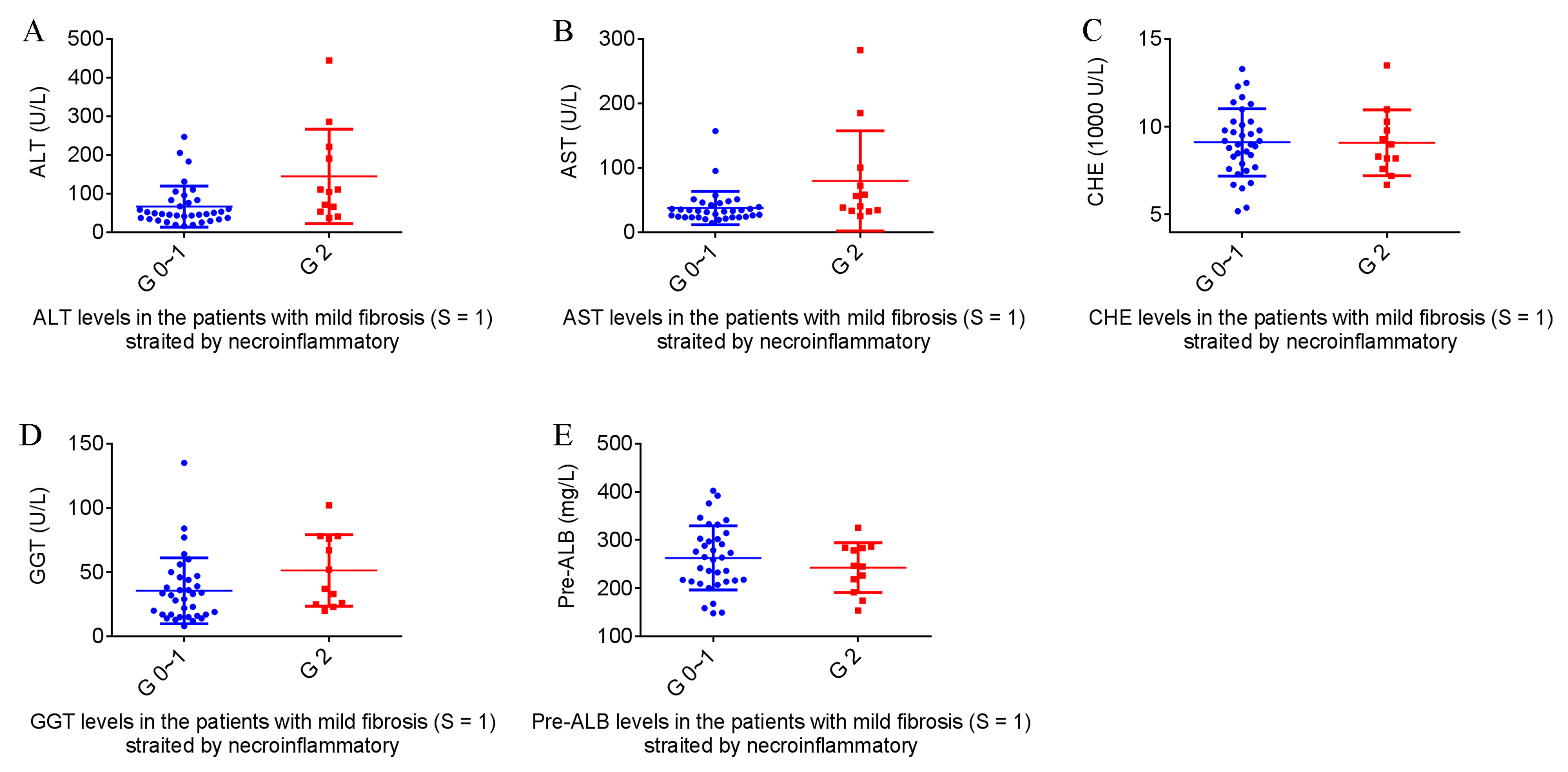

Supplement: Figure S3 — Levels of enrolled variables in the HBeAg(−) patients with S1 fibrosis with no to mild or moderate inflammation in the training set. (A) ALT (Mann Whitney test, p = 0.0070); (B) AST (Mann Whitney test, p = 0.0066); (C) CHE (Mann Whitney test, p = 0.7717); (D) GGT (Mann Whitney test, p = 0.0464); (E) pre-ALB (Mann Whitney test, p = 0.3470). Abbreviations: ALT, alanine aminotransferase; AST, aspartate aminotransferase; CHE, cholinesterase; GGT, γ-glutamyl transpeptidase; pre-ALB, pre-albumin. (TIF) [file pone.0111641.s003.tif]
