# Supplementary material for: Liver Stiffness Measurement-Based Scoring System for Significant Inflammation Related to Chronic Hepatitis B
Source: PLoS One. 2014 Oct 31;9(10):e111641. doi: 10.1371/journal.pone.0111641 (PMC4216134; doi:10.1371/journal.pone.0111641)
Supplement: Table S1 — Area under the curve for differentiating specific stage of fibrosis (S). (DOCX) [file pone.0111641.s004.docx]

## SUPPLEMENTARY MATERIAL

**Table S1.** Area under the curve for differentiating specific stage of fibrosis (S)

| S2 | S3 | S4 | Reference |
| --- | --- | --- | --- |
| 0.80 | 0.90 | 0.96 | *Gut* 2006; 55: 403 |
| 0.84 | 0.89 | 0.94 | *Gastroenterology* 2008; 134: 960 |
| 0.87 (S ≥ 2) | NA | 0.96 | *Clin Gastroenterol Hepatol* 2007; 5: 1214 |
| 0.88 (S ≥ 2) | NA | 0.99 | *Ultrasound Med Biol* 2003; 29: 1705 |
| NA | NA | 0.95 | *Hepatology* 2006; 44: 1511 |
