# Supplementary material for: Liver Stiffness Measurement-Based Scoring System for Significant Inflammation Related to Chronic Hepatitis B
Source: PLoS One. 2014 Oct 31;9(10):e111641. doi: 10.1371/journal.pone.0111641 (PMC4216134; doi:10.1371/journal.pone.0111641)
Supplement: Table S2 — Characteristics of the patients enrolled in the training and validation sets. (DOCX) [file pone.0111641.s005.docx]

## SUPPLEMENTARY MATERIAL

Table S2. Characteristics of the patients enrolled in the training and validation sets

| Patient group | Training set (n = 327) | | Validation set (n = 106) | |
| --- | --- | --- | --- | --- |
|  | HBeAg(+)  (n = 190) | HBeAg(-)  (n = 137) | HBeAg(+)  (n = 61) | HBeAg(-)  (n = 45) |
| Age, yr | 32.5 ± 8.6 | 40.4 ± 9.7 | 31.3 ± 8.4 | 39.4 ± 8.8 |
| Male, n (%) | 144 (75.8) | 111 (81.0) | 45 (73.8) | 41 (91.1) |
| ALT, IU/L | 175.7 ± 240.6 | 101.9 ± 149.2 | 208.4 ± 242.1 | 164.3 ± 202.7 |
| Normal ALT, n (%) | 19 (10.0) | 41 (29.9) | 7 (11.5) | 8 (17.8) |
| AST, IU/L | 93.7 ± 138.5 | 56.0 ± 69.1 | 124.4 ± 147.3 | 82.8 ± 93.5 |
| Normal AST, n (%) | 69 (36.3) | 85 (59.9) | 19 (31.1) | 15 (33.3) |
| CHE, IU/L | 8200 ± 1700 | 8600 ± 1800 | 7600 ± 1700 | 7600 ± 2100 |
| GGT, IU/L | 52.6 ± 48.8 | 48.5 ± 53.9 | 62.1 ± 73.4 | 83.2 ± 75.9 |
| Globulin, g/L | 28.3 ± 4.0 | 27.6 ± 4.3 | 30.8 ± 5.5 | 32.3 ± 5.1 |
| Albumin, g/L | 44.6 ± 3.2 | 44.4 ± 3.1 | 42.9 ± 3.7 | 42.0 ± 4.0 |
| Pre-Albumin, mg/L | 222.0 + 68.1 | 240.5 ± 66.9 | 199.7 ± 64.3 | 195.1 ± 66.0 |
| **Liver histology**^*^ |  |  |  |  |
| Activity, n (%) |  |  |  |  |
| G0 | 5 (2.6) | 12 (8.8) | 5 (8.2) | 1 (2.2) |
| G1 | 67 (35.3) | 62 (45.2) | 12 (19.7) | 6 (13.3) |
| G2 | 81 (42.6) | 43 (31.4) | 23 (37.7) | 17 (37.8) |
| G3 | 35 (18.4) | 20 (14.6) | 18 (29.5) | 20 (44.4) |
| G4 | 2 (1.1) | 0 (0) | 3 (4.9) | 1 (2.2) |
| Fibrosis, n (%) |  |  |  |  |
| S0 | 19 (10.0) | 31 (22.6) | 3 (4.9) | 1 (2.2) |
| S1 | 86 (45.3) | 47 (34.3) | 26 (42.5) | 15 (33.4) |
| S2 | 50 (26.3) | 31 (22.6) | 14 (23.0) | 12 (26.7) |
| S3 | 31 (16.3) | 18 (13.1) | 14 (23.0) | 11 (24.4) |
| S4 | 4 (2.1) | 10 (7.3) | 4 (6.6) | 6 (13.3) |
| HBV DNA, log_10_ IU/ml | 7.04 ± 1.51 | 4.60 ± 1.58 | 7.26 ± 1.31 | 5.34 ± 1.83 |

^*^According to the Scheuer scoring system.

ALT, alanine aminotransferase; AST, aspartate aminotransferase; CHE, cholinesterase; GGT, γ-glutamyl transpeptidase.
