# Supplementary material for: Liver Stiffness Measurement-Based Scoring System for Significant Inflammation Related to Chronic Hepatitis B
Source: PLoS One. 2014 Oct 31;9(10):e111641. doi: 10.1371/journal.pone.0111641 (PMC4216134; doi:10.1371/journal.pone.0111641)
Supplement: Table S3 — The Gini index of the factors associated with significant inflammation (G). (DOCX) [file pone.0111641.s006.docx]

## SUPPLEMENTARY MATERIAL

**Table S3.** The Gini index of the factors associated with significant inflammation (G)

|  | HBeAg(+) | HBeAg(-) |
| --- | --- | --- |
| Factor | Mean Decrease Gini index | Mean Decrease Gini index |
| Stage of fibrosis | 27.394 | 32.241 |
| Pre-Albumin | 16.248 | 23.893 |
| GGT | 15.652 | 22.763 |
| AST | 9.923 | 14.701 |
| Cholinesterase | 9.411 | 24.526 |
| ALT | 8.730 | 9.560 |
| Albumin | 8.043 | 18.462 |
| Globulin | 6.425 | 7.423 |
| HBV DNA, log_10_ IU/ml | 4.706 | 5.178 |
