# Supplementary material for: Liver Stiffness Measurement-Based Scoring System for Significant Inflammation Related to Chronic Hepatitis B
Source: PLoS One. 2014 Oct 31;9(10):e111641. doi: 10.1371/journal.pone.0111641 (PMC4216134; doi:10.1371/journal.pone.0111641)
Supplement: Table S4 — Calculation of Fibrosis-based activity score. (DOCX) [file pone.0111641.s007.docx]

## SUPPLEMENTARY MATERIAL

**Table S4.** Calculation of Fibrosis-based activity score

| Variable |  |  |  |  |
| --- | --- | --- | --- | --- |
| HBeAg(+) | AUC | 1/(1-AUC) | 1/(1-AUC)/4.0 | 1/(1-AUC)/4.0*10 |
| Fibrosis | 0.890 | 9.1 | 2.3 | 23 |
| Pre-albumin | 0.819 | 5.5 | 1.4 | 14 |
| GGT | 0.806 | 5.2 | 1.3 | 13 |
| AST | 0.752 | 4.0 | 1.0 | 10 |
| CHE | 0.747 | 4.0 | 1.0 | 10 |
| HBeAg(-) | AUC | 1/(1-AUC) | 1/(1-AUC)/4.9 | 1/(1-AUC)/4.9*10 |
| Fibrosis | 0.917 | 12.0 | 2.4 | 24 |
| CHE | 0.870 | 7.7 | 1.6 | 16 |
| Pre-albumin | 0.869 | 7.6 | 1.6 | 16 |
| GGT | 0.865 | 7.4 | 1.5 | 15 |
| Albumin | 0.826 | 5.7 | 1.2 | 12 |
| AST | 0.797 | 4.9 | 1.0 | 10 |
