# Supplementary material for: Liver Stiffness Measurement-Based Scoring System for Significant Inflammation Related to Chronic Hepatitis B
Source: PLoS One. 2014 Oct 31;9(10):e111641. doi: 10.1371/journal.pone.0111641 (PMC4216134; doi:10.1371/journal.pone.0111641)
Supplement: Table S5 — Diagnostic performance of logistic regression and random forest for recognizing significant inflammation. (DOCX) [file pone.0111641.s008.docx]

## SUPPLEMENTARY MATERIAL

**Table S5.** Diagnostic performance of logistic regression and random forest for recognizing significant inflammation

|  |  | HBeAg(+) Training set | HBeAg(-) Training set |
| --- | --- | --- | --- |
| Logistic regression | AUC | 0.945 | 0.982 |
|  | Sensitivity (%) | 90.7 | 96.0 |
|  | Specificity (%) | 89.5 | 97.4 |
|  | PPV (%) | 86.0 | 94.1 |
|  | NPV (%) | 93.2 | 98.3 |
| Random forest | AUC | 0.978 | 0.982 |
|  | Sensitivity (%) | 95.4 | 96.0 |
|  | Specificity (%) | 93.4 | 97.4 |
|  | PPV (%) | 91.2 | 94.1 |
|  | NPV (%) | 96.6 | 98.3 |
