# Supplementary material for: Liver Stiffness Measurement-Based Scoring System for Significant Inflammation Related to Chronic Hepatitis B
Source: PLoS One. 2014 Oct 31;9(10):e111641. doi: 10.1371/journal.pone.0111641 (PMC4216134; doi:10.1371/journal.pone.0111641)
Supplement: Table S6 — Relationship between number of enrolled variables and diagnostic performance of the prediction score. (DOCX) [file pone.0111641.s009.docx]

## SUPPLEMENTARY MATERIAL

**Table S6.** Relationship between number of enrolled variables and diagnostic performance of the prediction score

| Factor | 7 variables | | 6 variables | | 5 variables | | 4 variables | |
| --- | --- | --- | --- | --- | --- | --- | --- | --- |
| HBeAg(+) | | | | | | | | |
| Training set | | | | | | | | |
| AUC | | 0.957 | | 0.959 | | 0.964 | | 0.962 |
| Cut-off | | 6 | | 11 | | 10 | | 11 |
| Sensitivity (%) | | 89.2 | | 94.6 | | 91.9 | | 97.2 |
| Specificity (%) | | 88.9 | | 88.9 | | 90.8 | | 87.6 |
| Validation set | | | | | | | | |
| AUC | | 0.972 | | 0.972 | | 0.971 | | 0.957 |
| Cut-off | | 6 | | 11 | | 10 | | 11 |
| Sensitivity (%) | | 95.2 | | 95.2 | | 90.5 | | 85.7 |
| Specificity (%) | | 90.0 | | 90.0 | | 92.5 | | 92.5 |
| HBeAg(-) | | | | | | | | |
| Training set | | | | | | | | |
| AUC | | 0.969 | | 0.978 | | 0.979 | | 0.978 |
| Cut-off | | 19 | | 12 | | 25 | | 8 |
| Sensitivity (%) | | 85.0 | | 85.0 | | 85.0 | | 100.0 |
| Specificity (%) | | 94.9 | | 94.0 | | 95.7 | | 88.9 |
| Validation set | | | | | | | | |
| AUC | | 0.983 | | 0.977 | | 0.969 | | 0.950 |
| Cut-off | | 19 | | 12 | | 25 | | 8 |
| Sensitivity (%) | | 95.2 | | 95.2 | | 85.7 | | 90.5 |
| Specificity (%) | | 95.8 | | 95.8 | | 100.0 | | 75.0 |
