# Supplementary material for: Liver Stiffness Measurement-Based Scoring System for Significant Inflammation Related to Chronic Hepatitis B
Source: PLoS One. 2014 Oct 31;9(10):e111641. doi: 10.1371/journal.pone.0111641 (PMC4216134; doi:10.1371/journal.pone.0111641)
Supplement: Table S7 — Efficacy of the fibrosis-based activity score in the patients with normal alanine aminotransferase. (DOCX) [file pone.0111641.s010.docx]

## SUPPLEMENTARY MATERIAL

**Table S7**. Efficacy of the fibrosis-based activity score in the patients with normal alanine aminotransferase

| Fibrosis-Activity Score | HBeAg (+) | | HBeAg (-) | |
| --- | --- | --- | --- | --- |
|  | Training set | Validation set | Training set | Validation set |
| Sensitivity (%) | NA^*^ | NA^*^ | 100.0 | 100.0 |
| Specificity (%) | 89.5 | 100.0 | 100.0 | 100.0 |
| PPV (%) | NA^*^ | NA^*^ | 100.0 | 100.0 |
| NPV (%) | 89.5 | 100.0 | 100.0 | 100.0 |

^*^ There were no patients with normal ALT have significant necroinflammation in both training set and validation set of the HBeAg(+) groups.
